# Fruiting bodies of the social amoeba *Dictyostelium discoideum* increase spore transport by *Drosophila*

**DOI:** 10.1186/1471-2148-14-105

**Published:** 2014-05-15

**Authors:** jeff smith, David C Queller, Joan E Strassmann

**Affiliations:** 1 Department of Biology, Washington University in St. Louis, St. Louis, MO 63130, USA

**Keywords:** Adaptation, Altruism, Cooperation, Dispersal

## Abstract

**Background:**

Many microbial phenotypes are the product of cooperative interactions among cells, but their putative fitness benefits are often not well understood. In the cellular slime mold *Dictyostelium discoideum*, unicellular amoebae aggregate when starved and form multicellular fruiting bodies in which stress-resistant spores are held aloft by dead stalk cells. Fruiting bodies are thought to be adaptations for dispersing spores to new feeding sites, but this has not been directly tested. Here we experimentally test whether fruiting bodies increase the rate at which spores are acquired by passing invertebrates.

**Results:**

*Drosophila melanogaster* accumulate spores on their surfaces more quickly when exposed to intact fruiting bodies than when exposed to fruiting bodies physically disrupted to dislodge spore masses from stalks. Flies also ingest and excrete spores that still express a red fluorescent protein marker.

**Conclusions:**

Multicellular fruiting bodies created by *D. discoideum* increase the likelihood that invertebrates acquire spores that can then be transported to new feeding sites. These results thus support the long-hypothesized dispersal benefits of altruism in a model system for microbial cooperation.

## Background

Microbes appear to cooperate to acquire resources, fend off competitors, survive harsh conditions, and disperse to new habitats [1]. We say "appear" because in many cases very little is known about how interactions observed in the laboratory actually function in their natural context. Small diffusible molecules might coordinate gene expression with local cell density, for example, or they might measure the local strength of diffusion [2,3]. Secondary metabolites might be antibiotics that kill off competitors, or they might be chemical signals among cooperators [4]. To understand the biological function of social interactions among microbes, we must understand how they affect fitness. Of the various phenotypic effects these traits have, which are favored by natural selection?

One model system for microbial cooperation is the cellular slime mold *Dictyostelium discoideum*[5]. These unicellular amoebae aggregate when starved and form multicellular fruiting bodies in which stress-resistant spores are held aloft by dead stalk cells—a form of microbial altruism. Fruiting bodies have long been hypothesized to be adaptations for dispersing spores to new feeding sites through contact with small soil invertebrates [6]. This hypothesis is consistent with several types of indirect evidence. Spore-bearing fruiting structures are common among soil-dwelling microbes as diverse as fungi and bacteria, and soil invertebrates can efficiently disperse microbes [7]. *Dictyostelium* aggregates migrate to soil surfaces using temperature, light, and gas cues [8], presumably to produce fruiting bodies where they are likely to contact passing invertebrates. In fruiting bodies, spores are suspended in a drop of liquid that is readily transferred upon contact. When co-housed with fruiting bodies, roaming soil invertebrates disrupt these spore masses [8]. The lack of genetic differentiation among North American populations of *D. discoideum*[9], suggests that spores do get efficiently dispersed. Wind is unlikely to contribute much: Dictyostelid spore masses dry into hard clumps not easily dislodged from stalks, while wind-dispersed microbes typically produce spores as a fine powder easily picked up by air currents.

There is no direct evidence that fruiting bodies actually increase dispersal, however. Invertebrate grooming might remove spores before they get dispersed. Or perhaps spores are just as easily acquired when invertebrates step on them. The biological function of fruiting bodies might instead be to provide protection from nematodes and fungi [5,9]. *Dictyostelium* is thus a model system for microbial cooperation in which the benefits of cooperation are poorly understood. Here, we attempt to remedy this awkward situation by experimentally testing whether fruiting bodies increase the likelihood that spores are picked up and carried by passing invertebrates—the first step in dispersal. The primary vectors of *D. discoideum* dispersal are unknown, so we exposed fruiting bodies to *Drosophila melanogaster* as an experimentally tractable stand-in and tested whether flies acquire spores more readily from intact than from disrupted fruiting bodies.

## Results

**Figure 1:**
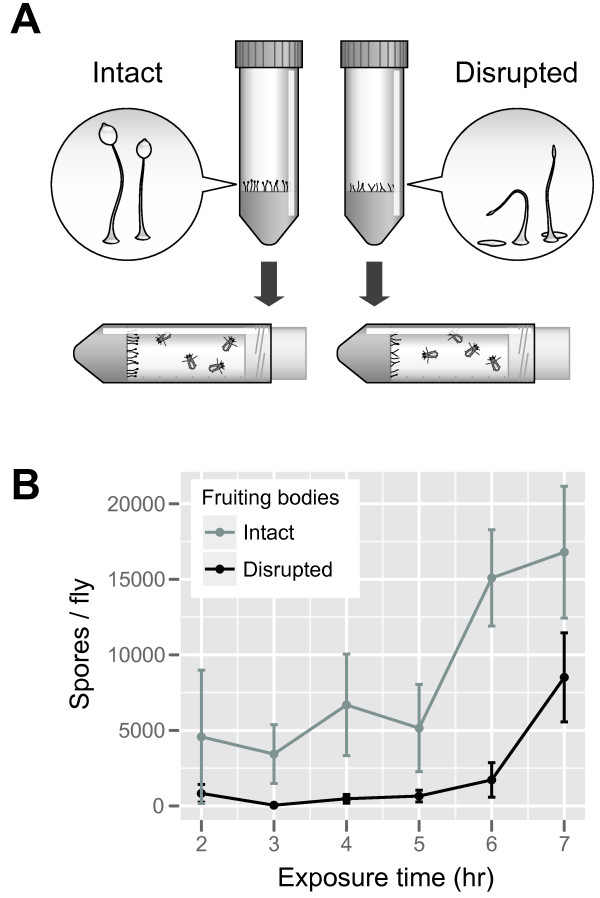
**Fruiting bodies created by social amoebae increase spore dispersal by arthropods. (A)** Dispersal assay. Amoebae created fruiting bodies on agar in the bottom of a conical tube. We disrupted fruiting bodies in some tubes by banging them several times against a hard surface, dislodging spore masses and causing fruiting bodies to fall over. We then turned tubes sideways and introduced fruit flies. At various times we sampled tubes and counted spores recovered from washed flies. **(B)** Flies pick up spores more readily from intact fruiting bodies. Data show mean ± SEM of 3–4 independent experimental replicates.

We exposed *D. melanogaster* either to intact fruiting bodies or to fruiting bodies physically disrupted to dislodge spore masses and collapse stalks (Figure 1A). During the assay we observed direct physical contact between flies and fruiting bodies when flies walked around inside tubes, often followed by grooming behavior. After exposure, we washed flies and counted recovered spores. Flies accumulated spores over time (Figure 1B; main effect of time *F*_1, 37_ = 20.4, *P* = 6.1 × 10^-5^). After 7 hrs of exposure to intact fruiting bodies, ~1% of the original cell population could be recovered from flies. Flies acquired spores more readily from intact than from disrupted fruiting bodies (Figure 1B; main effect of treatment *F*_1, 37_ = 17.1, *P* = 2.0 × 10^-4^), though they did accumulate spores in both treatments.

**Figure 2:**
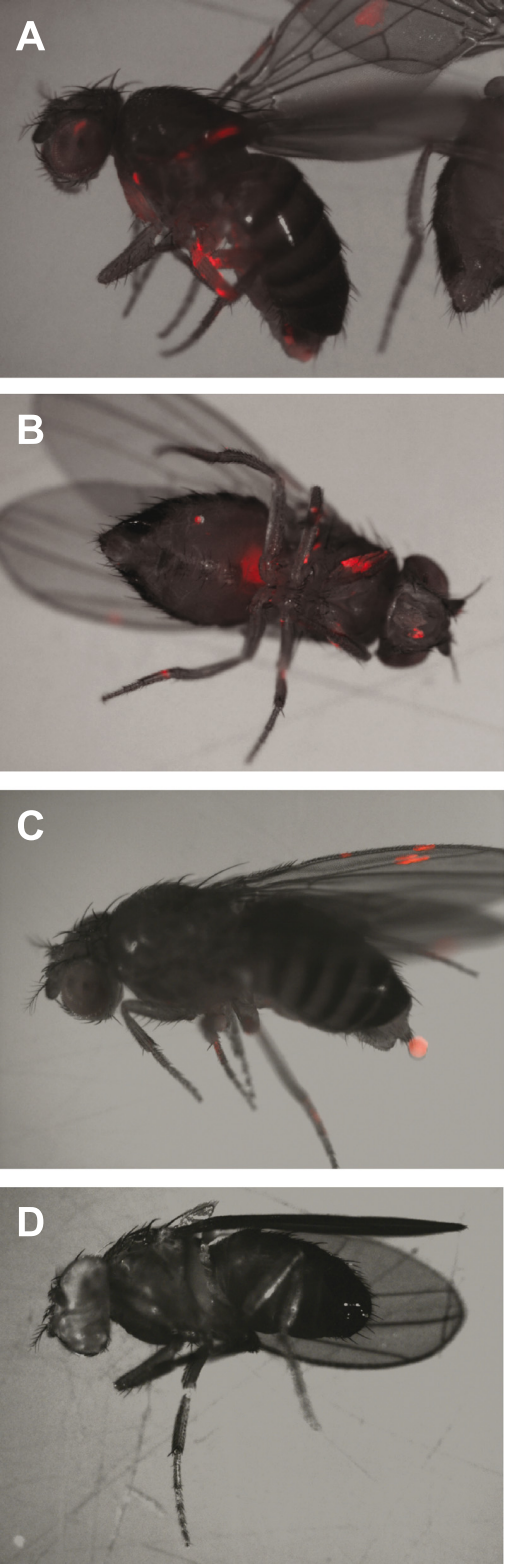
**Location of carried spores. (A-C)** Spores accumulated on *D. melanogaster* legs, wings, eyes, and mouthparts. Flies also ingested **(B)** and excreted **(C)** spores without degrading fluorescence. **(D)** Fly not exposed to spores. Images show composite of reflected light and red fluorescence.

To visualize where spores were on flies, we exposed flies to fruiting bodies of fluorescently labeled *D. discoideum*. We observed fluorescence on fly legs, wings, eyes, and mouthparts (Figure 2). In a few cases, we also observed fluorescence within fly abdomens (Figure 2B) and in fly excreta (Figure 2C).

## Discussion

To test the hypothesis that the fruiting bodies of social amoebae are cooperative adaptations for dispersal, we examined whether the structure of fruiting bodies increases their likelihood of being picked up by small invertebrates. We found that, indeed, *D. melanogaster* fruit flies more readily acquire *D. discoideum* spores from intact fruiting bodies than from fruiting bodies that have been knocked over or had their spore masses dislodged. Spores are thus more easily acquired from fruiting bodies than from agar or plastic surfaces. The effect is quite large, especially at the earliest time points—two-fold or more and thus more than enough to offset the expected cost of allocating ~20% of all cells to stalk. We did see flies groom themselves after contact with fruiting bodies. This may have decreased the number of spores they carried, but it was not enough to prevent fruiting bodies from increasing carriage altogether.

Fruiting bodies may increase spore transport by preventing them from sticking to soil surfaces, which only requires stalks be long enough to raise spore masses above the substrate. Indeed, several Dictyostelid species produce fruiting bodies only hundreds of microns long [10]. Fruiting bodies may also increase spore transport by raising them to increase contact with passing invertebrates. In this case, strains with longer stalks might have higher dispersal rates, though our data do not address this hypothesis. Multicellular development can also increase dispersal when species form slugs that migrate long distances [11], placing fruiting bodies in locations where they are more likely to come into contact with dispersers [8] or carrying amoebae to new food sources [12].

In addition to the spores on fly surfaces, we also observed flies ingest and excrete spores. Flies may have ingested spores by grooming after contacting fruiting bodies or by drinking from fluid droplets in spore masses (*Drosophila*, after all, means "dew lover"). We did not test whether excreted spores were viable, but we believe it likely. Viable Dictyostelids have been found in the guts of wild-caught pillbugs, earthworms, and ground-feeding songbirds [13,14]. Spores also survive passage through the guts of nematodes [9]. Yeast and bacteria survive passage through *Drosophila* guts [15]. It is unknown whether Dictyostelids produce compounds that attract dispersers, but they do have the genomic potential to produce many secondary metabolites [16]. Ingestion would put spores near dense populations of prey bacteria in guts and dung.

Many aspects of Dictyostelid natural history remain unknown, including how often cells form fruiting bodies, how cells disperse to new feeding sites, and what animal species are common dispersal vectors. In our experiments, we used *D. melanogaster* as a tractable model for any arthropod that moves around, contacts spores, and grooms. *Drosophila* are not commonly found associated with soil or dung, so it seems unlikely that they disperse social amoebae very often in natural habitats. Further work is needed to identify the primary vectors of Dictyostelid spores and test whether our experimental results generalize to natural habitats and dispersers.

## Conclusions

While many microbial phenotypes appear to be the product of cooperative interactions among cells, understanding the biological function of these traits requires that we understand how they contribute to microbial fitness. Here we have experimentally tested the long-standing hypothesis that multicellular fruiting bodies produced by the social amoeba *Dictyostelium discoideum* are cooperative adaptations that increase dispersal via passing invertebrates. Using *Drosophila melanogaster* as a convenient model, our results show that the physical structure of fruiting bodies increases the likelihood of spore carriage by flies. These results thus support the long-hypothesized benefits of altruism in a model system for microbial cooperation.

## Methods

### Strains

*Dictyostelium discoideum* strain NC28.1 was originally collected in North Carolina [17]. NC28.1 *rfp* is a derivative engineered to express red fluorescent protein (*rfp*) [18]. We grew *D. discoideum* on a strain of *Klebsiella pneumoniae* bacteria (obtained from N. Buttery) with spontaneous resistance to the antimicrobial G418. We stored *D. discoideum* and *K. pneumoniae* strains at -80°C in 20% (v/v) glycerol. We obtained wild-type (Canton-S) *Drosophila melanogaster* from Y. Ben-Shahar (Washington University) and maintained them at 22°C in 200 ml vials containing 50 ml Formula 4–24 Instant *Drosophila* Medium (Carolina Biological Supply, Burlington NC, USA) with constant light.

### Dispersal assay

We tested how fruiting bodies affect dispersal by exposing flies to either intact fruiting bodies or to fruiting bodies disrupted so that most spores rested on the substrate instead of at the end of upright stalks (Figure 1). To obtain fresh spores with which to start experiments, we allowed amoebae to grow and develop on *K. pneumoniae* lawns on 2.0% (weight/volume) agar plates of SM medium (Formedium, Hunstanton, United Kingdom) at 22°C. We dislodged spore masses from fruiting bodies by banging plates upside down, harvested spores from the plate lid with 1.0 ml KK2 buffer (per liter: 2.25 g KH_2_HPO_4_, 0.67 g K_2_HPO_4_), and resuspended them in KK2 to 1 × 10^7^ cells/ml using spore counts in a hemacytometer.

To obtain *D. discoideum* fruiting bodies, we first plated 10^6^ *D. discoideum* spores onto SM plates with 100 μl of stationary phase *K. pneumoniae* culture grown for 2 days in SM broth at 22°C without shaking. We incubated plates for 2 days at 22°C with passive humidity and overhead light. We harvested log-phase *D. discoideum* cells off these plates with an ethanol-sterilized plastic spatula and resuspended them in 12 ml cold KK2. We centrifuged cells for 3 min at 300 × g, washed them three times in 12 ml cold KK2, and resuspended them to 1.0 × 10^8^ cells/ml using spore counts in a hemacytometer. We deposited 10^7^ cells (100 μl) onto 10 ml KK2 agar (2.0% w/v) in the bottom of a 50 ml conical tube. We incubated these tubes 4 days at 22°C with ambient humidity and overhead light, during which time *D. discoideum* cells created fruiting bodies.

We left fruiting bodies intact in control tubes. For the experimental treatment, we disrupted fruiting bodies by banging conical tubes onto a hard surface several times, causing fruiting bodies to fall over and/or spore masses to fall onto the surface of the agar. We anesthetized adult flies with FlyNap (Carolina Biological Supply, Burlington NC, USA), introduced 5 males and 5 females into each tube, and let the tubes sit sideways without further disturbance. We kept conical tubes sideways so that flies would need to walk into or fly into fruiting bodies, rather than just fall onto them. To sample the flies in a tube, we anesthetized flies, collected them together into 250 μl KK2 buffer supplemented with 0.1% NP-40 detergent, vortexed the tube for ~20 seconds, and determined the density of recovered spores recovered using counts in a hemacytometer. We replicated these experiments using bacteria and amoebae independently grown from frozen stocks on different days.

We analyzed data by fitting generalized linear models to spore count data in *R* v2.15.3 using the *glm* command. Data were overdispersed, so we used quasipoisson errors with a log link function. We accounted for variation in the number of flies and hemacytometer area counted using the *offset* command. Because each data point came from a separate tube (destructive sampling), we modeled time as a fixed effect. We tested the significance of model terms by performing *F* tests on models fit with and without the term of interest.

### Microscopy

To visualize spores on flies, we exposed 10 flies to fruiting bodies of NC28.1 *rfp* for 24 hr as described above, except to maximize fluorescence signal we supplemented SM media with 5 μg/ml of the aminoglycoside antibiotic G418 to select against nonfluorescent mutants that appear during cell culture. After exposure, we anaesthetized flies and incubated them 20 min at -20°C. We acquired reflected light and red fluorescent images of flies using a Zeiss SV11 stereomicroscope (Carl Zeiss Microscopy LLC., Thornwood NY, USA) and assembled composite images using Adobe Photoshop (Adobe Systems, San Jose CA, USA).

## Availability of supporting data

The data set supporting the results of this article is available in the Dryad repository at doi:10.5061/dryad.9ht88 [19].

## Competing interests

The authors declare that they have no competing interests.

## Authors’ contributions

js conceived the study. All authors contributed to experimental design. js performed the experiments, analyzed the data, created the figures, and wrote the paper. DCQ and JES provided feedback on manuscript drafts. All authors read and approved the final manuscript.
